# Enhancing the Efficacy of Poly‐l‐Lactic Acid Injections With Micro‐Focused Ultrasound: An Evaluation of Combined Treatment and Optimal Sequence

**DOI:** 10.1111/jocd.70637

**Published:** 2026-01-14

**Authors:** Changxin Jin, Dan Li, Wei Zhan, Shixu Wang, Gaoxu Li, Ruixue Li, Yijie Wang, Bo Zhou, Fulin Chen, Qiong Wu

**Affiliations:** ^1^ Department of Plastic and Reconstructive Surgery, Xijing Hospital Air Force Medical University Xi'an China; ^2^ The First Affiliated Hospital of Northwest University· Xi'an No. 1 Hospital Xi'an China; ^3^ Liudalie Medical Group Guangzhou China; ^4^ Provincial Key Laboratory of Biotechnology of Shaanxi, Key Laboratory of Resource Biology and Modern Biotechnology in Western China, Faculty of Life Science Northwest University Xi'an China

**Keywords:** combination therapy, micro‐focused ultrasound, poly‐l‐lactic acid, skin rejuvenation, type III collagen

## Abstract

**Introduction:**

Poly‐l‐lactic acid (PLLA) and micro‐focused ultrasound (MFU) are widely used for skin rejuvenation, each promoting collagen remodeling through distinct mechanisms. While combined use is common in practice, the optimal treatment sequence and safety profile remain unclear. This study aimed to evaluate the synergistic effects and ideal sequencing of PLLA and MFU in a preclinical model to guide clinical protocols.

**Methods:**

Three Bama miniature pigs were subjected to six treatment conditions on demarcated abdominal skin zones: (a) PLLA alone, (b) MFU alone, (c) MFU immediately before PLLA, (d) MFU 1 month before PLLA, (e) MFU 1 month after PLLA, and (f) untreated control. Löviselle PLLA was injected subcutaneously, while MFU was administered using the MFUS Pro system. Skin biopsies were collected at day 180. Histological (Masson's trichrome, Sirius Red, Verhoeff–Van Gieson, H&E), SEM, and quantitative image analyses were performed to assess dermal thickness, collagen subtype distribution, elastin regeneration, fat septa organization, and PLLA microsphere stability.

**Results:**

PLLA alone increased dermal thickness by 23.7% and raised the type III/I collagen ratio to 0.79 ± 0.06 (*p* < 0.01). MFU alone mainly enhanced type I collagen (ratio: 0.39 ± 0.04) and improved fiber alignment. The combined treatment with MFU immediately before PLLA achieved the best outcomes: dermal thickness increased by 35.2%, and the type III/I collagen ratio reached 0.92 ± 0.05 (both *p* < 0.01). This group also showed denser elastin fibers, well‐structured fat septa, and the most compact collagen morphology under SEM. MFU did not accelerate PLLA degradation or increase inflammation, with no significant difference in microsphere size or inflammatory infiltration compared to PLLA alone (*p* > 0.05).

**Conclusion:**

Sequential application of MFU immediately before PLLA enhances dermal remodeling more effectively than either treatment alone, without compromising PLLA microsphere stability. These findings support the clinical potential and safety of this combinatory approach, offering evidence‐based guidance for optimizing skin rejuvenation protocols.

## 1 Introduction

Skin laxity is a common concern associated with aging, characterized by a decline in dermal elasticity, collagen production, and dermal thickness. Effective treatment strategies often target the stimulation of collagen synthesis to restore skin tightness and elasticity. Among these, Poly‐l‐lactic acid (PLLA), a medical device, has emerged as a well‐established method for addressing skin laxity through gradual and sustained collagen production [1]. PLLA's biocompatibility and ability to induce structural improvements make it a popular choice in aesthetic procedures [2].

Micro‐focused ultrasound (MFU) is a traditional non‐invasive skin tightening treatment that delivers targeted ultrasound energy to the dermis and subcutaneous tissues, creating thermal coagulation points. These thermal effects stimulate collagen remodeling and elastin production, improving skin firmness and elasticity. Its ability to target specific skin layers, including the superficial muscular aponeurotic system (SMAS), has made it a widely used modality for addressing skin laxity [3].

While PLLA and MFU are effective as standalone treatments, their combination is increasingly employed in clinical practice to achieve enhanced outcomes. PLLA provides gradual and sustained structural support through collagen synthesis, while MFU offers immediate tightening and elastin induction. Similar synergistic effects have been observed in studies combining energy‐based devices with injectable treatments [4]. For example, combining MFU‐V with hyperdilute CaHA‐CMC injections significantly improved skin laxity, with the sequence of MFU‐V followed by CaHA‐CMC yielding superior outcomes in elastin synthesis and patient satisfaction [5]. Another study on monopolar RF treatment over fillers reported enhanced fibroplasia and collagen deposition when combined with injectable fillers like PLLA and CaHA [6]. Additionally, a study demonstrated that combining fractional CO_2_ laser with hyaluronic acid injections significantly improved skin texture and hydration. The fractional CO_2_ laser was found to enhance hyaluronic acid diffusion and stimulate collagen production [7]. Furthermore, the combination of Q‐switched Nd:YAG laser with photoacoustic pulse technology and polynucleotide (PN) salmon DNA injections significantly improved skin brightness, pigmentation, and elasticity, demonstrating its potential as an effective approach for facial photo‐rejuvenation [8].

Despite the positive outcomes reported with various combined treatments, concerns remain in clinical practice regarding the potential structural disruption caused by energy‐based devices to degradable collagen biostimulatory agents, which could compromise their therapeutic efficacy [9]. Therefore, confirming the safety, effectiveness, and optimal sequencing of combining mainstream collagen biostimulatory agents like PLLA with traditional modalities such as MFU is of paramount importance. Addressing these questions will help refine clinical protocols and ensure consistent, reliable outcomes.

By systematically analyzing the effects of different treatment sequences in a preclinical animal model, this research aims to provide foundational evidence for the combined use of PLLA and MFU. The findings are intended to guide future clinical protocols, optimizing outcomes for patients seeking improved skin laxity and elasticity.

## 2 Materials and Methods

### 2.1 Experimental Animals and Experimental Plan

Three castrated male Bama miniature pigs, aged 6–8 months and weighing approximately 45 kg each, were selected as the animal models for this study. The skin of Bama miniature pigs is highly similar to human skin in terms of dermal thickness, fibroblast density, collagen composition, and elastin distribution. These three pigs were used as parallel experimental subjects to ensure the reproducibility and reliability of the results. All animals were housed under standard laboratory conditions, with an environmental temperature maintained at 18°C–24°C and a 12‐h light/dark cycle [10]. Adequate food and water were provided throughout the study, with weight gain controlled to < 5%. Monthly monitoring of body temperature was conducted to confirm the animals' health status. All experimental protocols were reviewed and approved by the Institutional Animal Care and Use Committee of the College of Life Sciences, Northwest University (Approval No. IACUC‐20241487), and all procedures conformed to the relevant national and institutional guidelines for the ethical treatment of laboratory animals.

**FIGURE 1:**
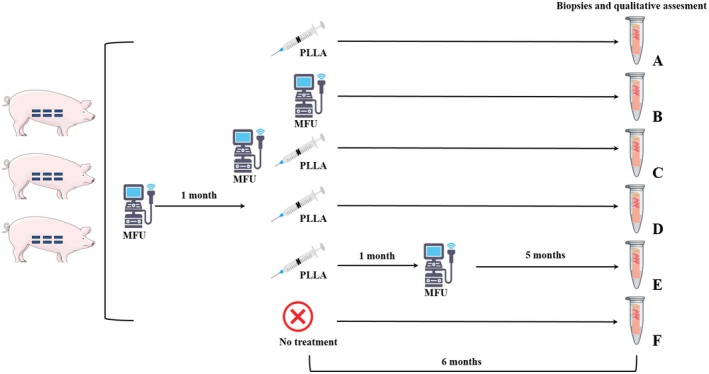
Schematic for the study design employed in the present study. The bilateral abdominal skin of each pig was divided into six regions, each measuring 5 × 5 cm, and randomly assigned to one of six treatment groups: (a) PLLA injection only; (b) MFU treatment only; (c) MFU treatment immediately before PLLA injection; (d) MFU treatment 1 month before PLLA injection; (e) MFU treatment 1 month after PLLA injection; (f) No treatment.

The bilateral abdominal skin of each pig was divided into six regions, each measuring 5 × 5 cm, and each region was randomly assigned using a computer‐generated random number sequence to one of six treatment groups (Figure 1). Prior to treatment, all pigs were anesthetized with pentobarbital. For PLLA treatment, a vial of 170 mg of Löviselle PLLA (Changchun Sinobiomaterials Co. Ltd.), synthesized using the proprietary PLLA‐LaSynPr technology, was reconstituted with 2 mL of saline and injected subcutaneously using a blunt cannula in a planar distribution. MFU treatment was performed using a micro‐focused ultrasound device, MicroUltra (manufacturer: Shenzhen Peninsula Medical Technology Co. Ltd.), equipped with an MFUS‐D 3.0 probe. According to the manufacturer's protocol, the device was operated in sliding mode for 3 min per treatment area, with conductive gel applied before treatment.

Body weight and temperature were measured on days 0, 30, 60, 90, 120, 150, and 180 post‐treatment. On day 180, skin samples, including the epidermis, dermis, and subcutaneous tissue, were collected from each treatment region using a 5 mm biopsy punch [11].

### 2.2 Histological Methods

Collected skin samples were fixed in 4% paraformaldehyde at room temperature for 48 h, followed by dehydration through a graded ethanol series (70%–99%) and clearing with a xylene series. The samples were then embedded in paraffin and sectioned into continuous slices with a thickness of 3–5 μm using a microtome [12]. For dermal thickness measurement, three vertical lines were randomly selected at specific intervals on each section under 4× magnification, and the distance between the basal layer and the adipose layer was measured along each line. The average dermal thickness for each treatment group was calculated by averaging the measurements of the three pigs in the same group [13]. The change ratio of dermal thickness was then determined by comparing the treatment groups to the untreated baseline group. Collagen quantification was performed using ImageJ software (National Institutes of Health, Bethesda, Maryland) [14]. Collagen density was analyzed using semi‐automated segmentation within the HSB (hue‐saturation‐brightness) color space. The collagen density was expressed as the area proportion of collagen‐positive pixels on sections at 20× magnification, with values ranging from 0% to 100% [15]. All histological slides were coded and analyzed in a blinded manner to minimize observer bias and ensure methodological rigor.

### 2.3 Histological Staining Analysis

Masson's trichrome staining was performed to evaluate the regeneration of collagen fibers, with collagen stained blue, muscle fibers red, and nuclei black, providing a detailed assessment of connective tissue repair. Sirius Red staining under polarized light was utilized to quantify type I collagen (red) and type III collagen (green), with Image J software used to calculate the ratio of type III to type I collagen. Verhoeff–Van Gieson staining was employed to qualitatively analyze elastin fibers, allowing the evaluation of their density and structural integrity in the treated tissues. HE staining was conducted to assess whether MFU treatment accelerated the degradation of subcutaneously injected PLLA microspheres. The quantitative results of microsphere distribution across different particle size ranges were obtained as the average from three Bama miniature pigs.

### 2.4 Scanning Electron Microscopy Analysis

Skin samples were prepared for scanning electron microscopy (SEM) by fixation in 2.5% glutaraldehyde at 4°C for 24 h, followed by dehydration through a graded ethanol series (50%–100%) and sputter‐coating with a thin layer of gold to enhance conductivity. SEM imaging was performed using a Helios system at 250× magnification with a 5 kV accelerating voltage [16]. The analysis focused on collagen fiber morphology, surface texture, and structural changes across different treatment groups, providing detailed insights into the effects of the treatments on tissue ultrastructure.

### 2.5 Statistical Analysis

All data were expressed as mean ± standard deviation (SD). Statistical analyses were performed using GraphPad Prism 9.0 (GraphPad Software, San Diego, CA, USA) [17]. One‐way analysis of variance (ANOVA) was used to compare differences among multiple groups, followed by *T*‐test for pairwise comparisons. Statistical significance was set at *p* < 0.05 and denoted with *, while highly significant differences were set at *p* < 0.01 and denoted with **.

## 3 Results

### 3.1 Physiological Monitoring of Study Objectives

Throughout the study, body temperature remained stable across all time points, ranging between 38.2°C and 38.5°C, indicating that the Bama mini pigs maintained a healthy physiological state. Body weight increased steadily, with a percentage increase of < 10% by Day 180 relative to baseline (Table 1). These moderate weight gains suggest that the body weight changes observed were within a normal growth range and are unlikely to have significantly influenced the study's results.

**TABLE 1:** Changes in body weight and temperature of Bama mini pigs during the study period.

Day	Temperature (°C)			Weight (kg)		
	Pig A	Pig B	Pig C	Pig A	Pig B	Pig C
0	38.2	38.3	38.4	44.0	44.8	44.5
30	38.3	38.4	38.2	44.5	45.2	45.3
60	38.5	38.3	38.3	45.8	46.5	46.2
90	38.3	38.2	38.4	46.5	47.0	46.8
120	38.4	38.4	38.5	47.2	47.7	47.6
150	38.2	38.5	38.3	47.8	48.2	48.1
180	38.3	38.3	38.3	48.3	48.7	48.5

### 3.2 Dermal Thickness

**FIGURE 2:**
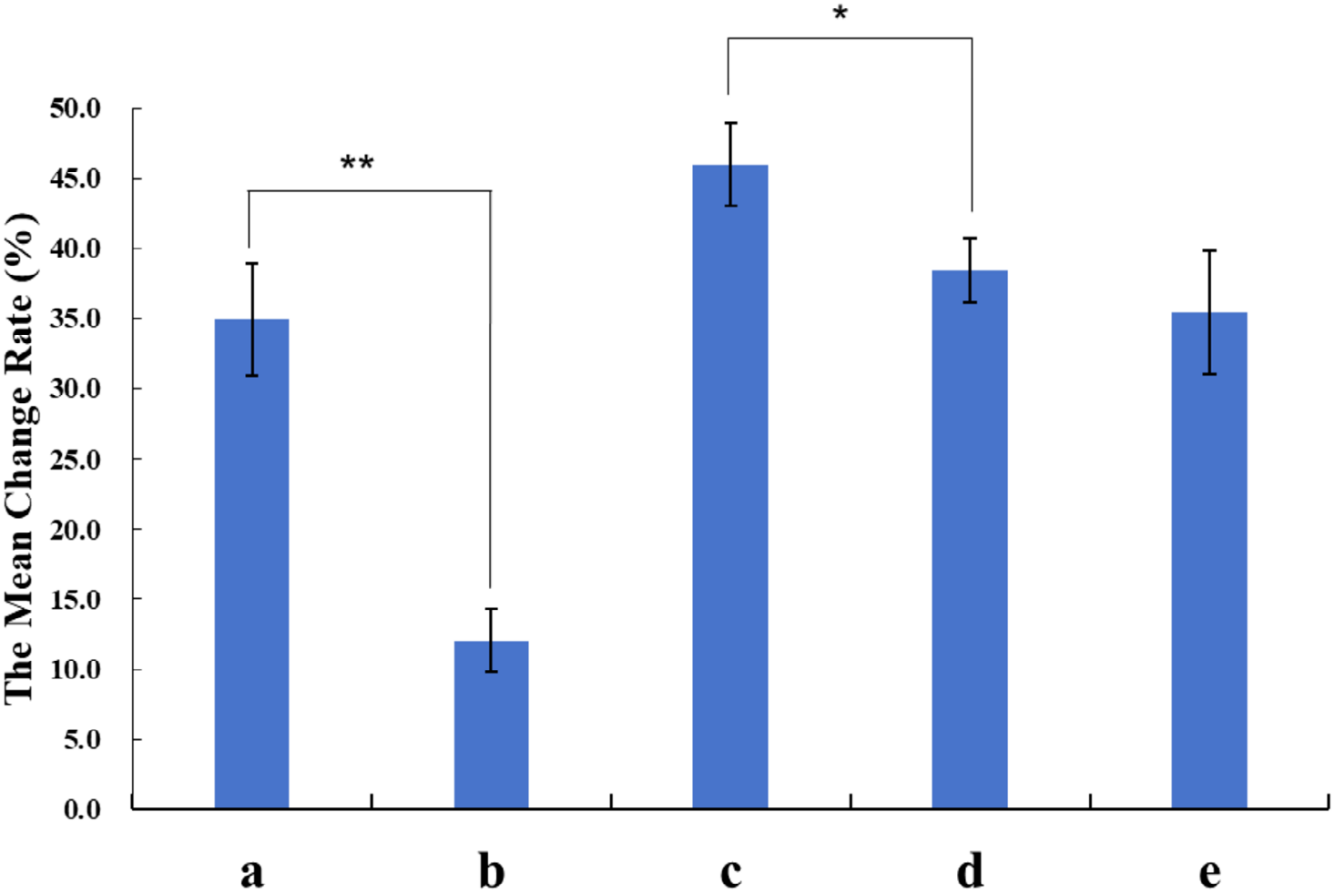
The mean change rate in dermal thickness for each experimental group compared to the untreated area 180 days post‐treatment. Group a: PLLA injection only; Group b: MFU treatment only; Group c: MFU treatment immediately before PLLA injection; Group d: MFU treatment 1 month before PLLA injection; Group e: MFU treatment 1 month after PLLA injection. Error bars represent the standard deviation (SD) of the measurements. Statistical significance is indicated as *p < 0.05 and **p < 0.01.

Histological analysis of dermal thickness revealed significant increases across all treatment groups compared to the control (Group f). PLLA injection alone (Group a) induced a moderate increase in dermal thickness, while MFU treatment alone (Group b) demonstrated a lesser effect. Notably, all PLLA‐containing groups (a, c, d, and e) showed significantly greater dermal thickening compared to the MFU‐only group (Group b) (*p* < 0.01). Among the combined treatment groups, Group c (MFU immediately prior to PLLA injection) resulted in the most significant increase in dermal thickness (*p* < 0.05) (Figure 2). These findings highlight that PLLA injection plays a dominant role in dermal thickening, and the combination with MFU further enhances this effect when administered sequentially.

### 3.3 Collagen Fiber Regeneration and Remodeling

**FIGURE 3:**
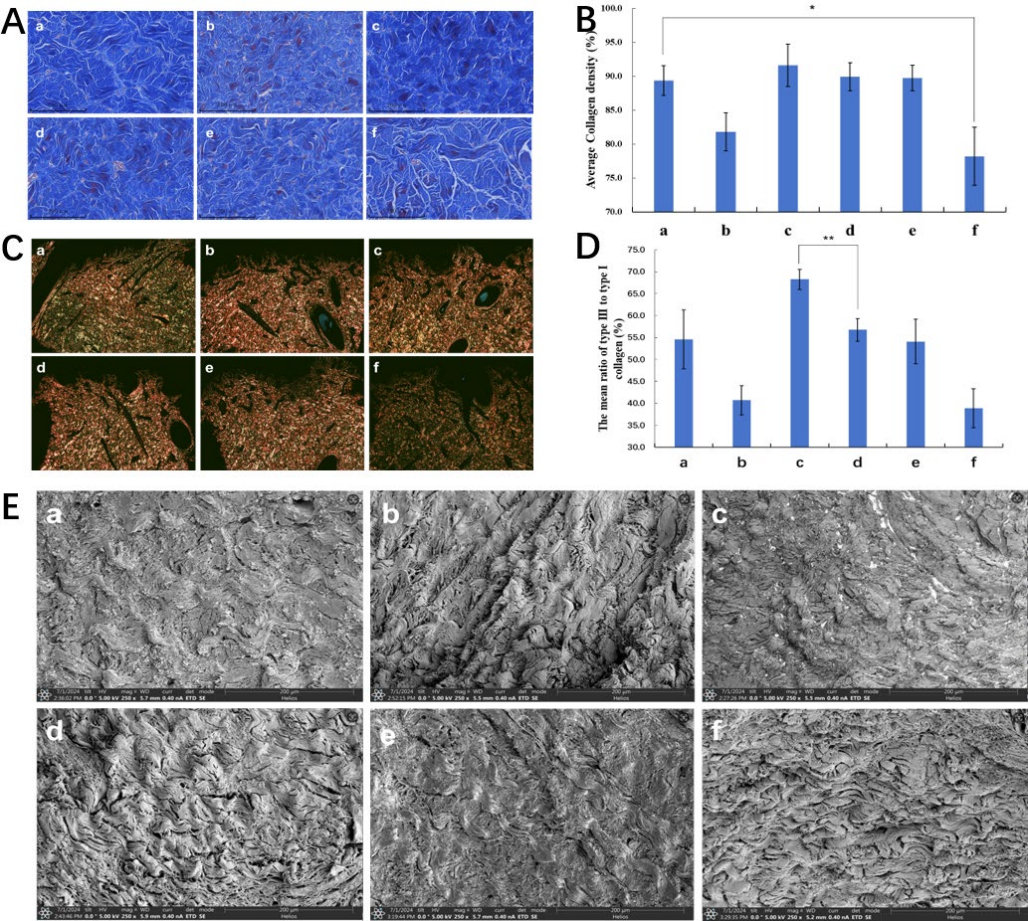
Collagen regeneration and remodeling in skin 180 days post‐treatment. (A) Masson's trichrome staining showing collagen distribution and structural organization across experimental groups. (B) Collagen density quantified in each group, demonstrating treatment‐induced changes. (C) Sirius Red staining under polarized light differentiating type I (red) and type III (green) collagen. (D) The ratio of type III to type I collagen analyzed quantitatively. (E) SEM images at 250× magnification highlighting collagen fiber morphology and organization. Groups include (a) PLLA injection only, (b) MFU treatment only, (c) MFU immediately before PLLA injection, (d) MFU one month before PLLA injection, (e) MFU one month after PLLA injection, and (f) no treatment. Quantitative analyses include error bars representing the standard deviation (SD), with statistical significance indicated as *p < 0.05 or **p < 0.01. All images were captured with a consistent field width of 200 μm for direct comparison.

Compared to the control group (Group f), MFU monotherapy (Group b) primarily optimized collagen fiber structure (Figure 3A), but its effect on promoting new collagen production was less pronounced. In contrast, PLLA‐containing groups (Group a, c, d and e) significantly increased collagen density, as evidenced by the stimulation of new collagen synthesis (*p* < 0.05) (Figure 3B). Sirius Red staining under polarized light revealed that MFU treatment (Group b) predominantly increased type I collagen, while PLLA (Group a) significantly upregulated type III collagen, which is typically finer and more delicate (Figure 3C). In the combination groups, particularly Group c (MFU immediately prior to PLLA), the ratio of type III to type I collagen was significantly elevated compared to Groups d, e, and the monotherapy groups (*p* < 0.01) (Figure 3D). Scanning electron microscopy (SEM) further provided detailed visualization of collagen fiber structure and morphology. In untreated controls (Group f), collagen fibers appeared sparse and disorganized. PLLA treatment (Group a) resulted in increased collagen density due to the stimulation of new type III collagen, leading to a visual impression of “finer fibers” and enhanced overall collagen content. Notably, Group c (MFU immediately prior to PLLA) displayed the most uniform, tightly packed, and well‐aligned collagen fibers, combining PLLA‐induced collagen density increases with the structural optimization effects of MFU. These findings suggest that the combined treatment achieves superior collagen remodeling (Figure 3E).

### 3.4 Subcutaneous Fat Septal Structure

**FIGURE 4:**
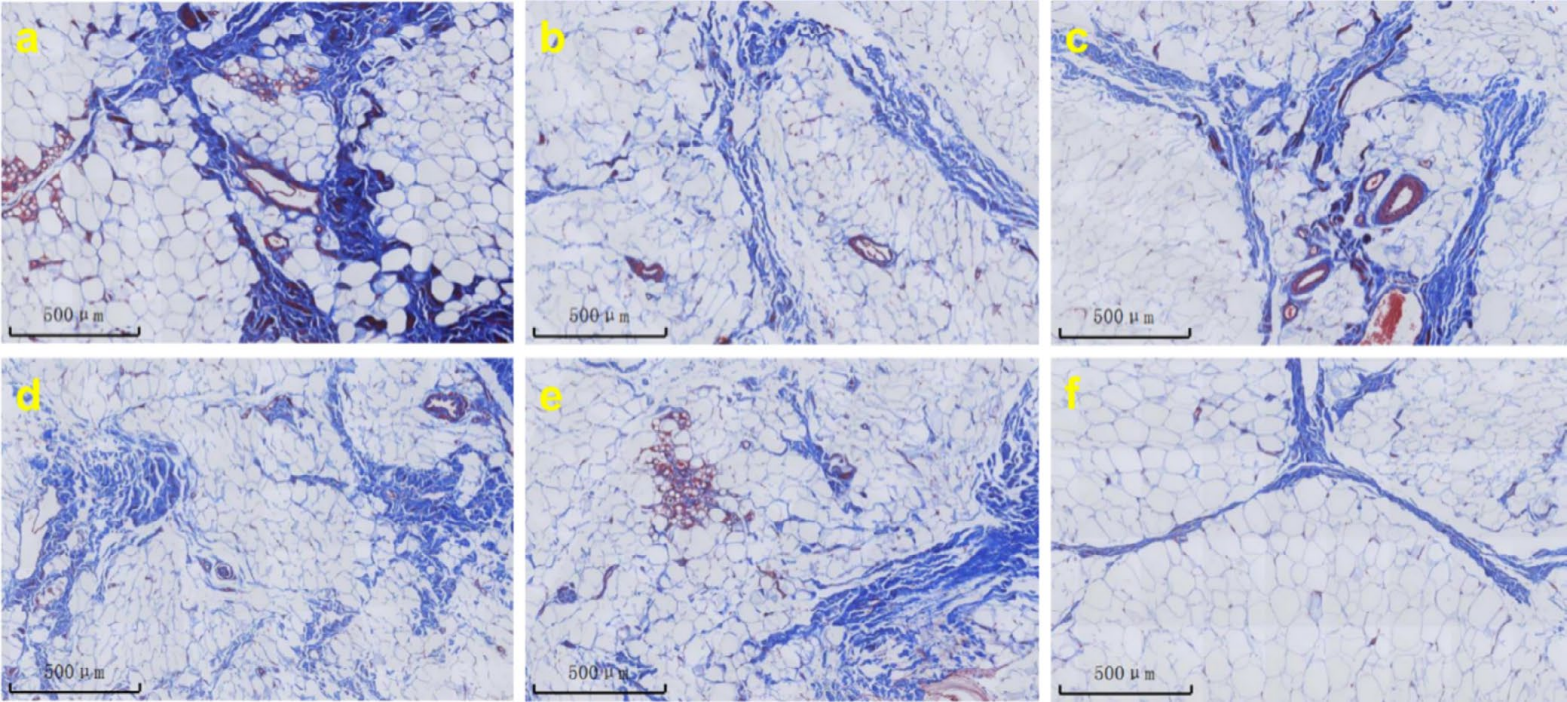
Representative images of subcutaneous adipose tissue and connective tissue stained with Masson's Trichrome from different experimental groups 180 days post‐treatment. (a) PLLA injection only; (b) MFU treatment only; (c) MFU treatment immediately before PLLA injection; (d) MFU treatment 1 month before PLLA injection; (e) MFU treatment 1 month after PLLA injection; (f) No treatment. Blue staining represents collagen fibers, while white spaces indicate adipose tissue vacuoles. Images were captured at a field width of 500 μm.

Masson's trichrome staining was used to evaluate the effects of different treatments on the subcutaneous fat layer, focusing on the fibrous septa. In the control group, the fat septa appeared thin, sparse, and disorganized, with minimal collagen content. PLLA treatment significantly increased septal thickness and collagen deposition, while MFU treatment primarily improved the alignment and organization of existing fibrous septa. The combination of MFU immediately prior to PLLA demonstrated the most pronounced improvements, with thickened, dense, and well‐organized fibrous septa, suggesting a synergistic effect between the two treatments (Figure 4). These findings align with the improvements observed in the dermis, highlighting that both PLLA and MFU exert complementary effects across multiple tissue layers.

### 3.5 Effects on Elastin Synthesis

**FIGURE 5:**
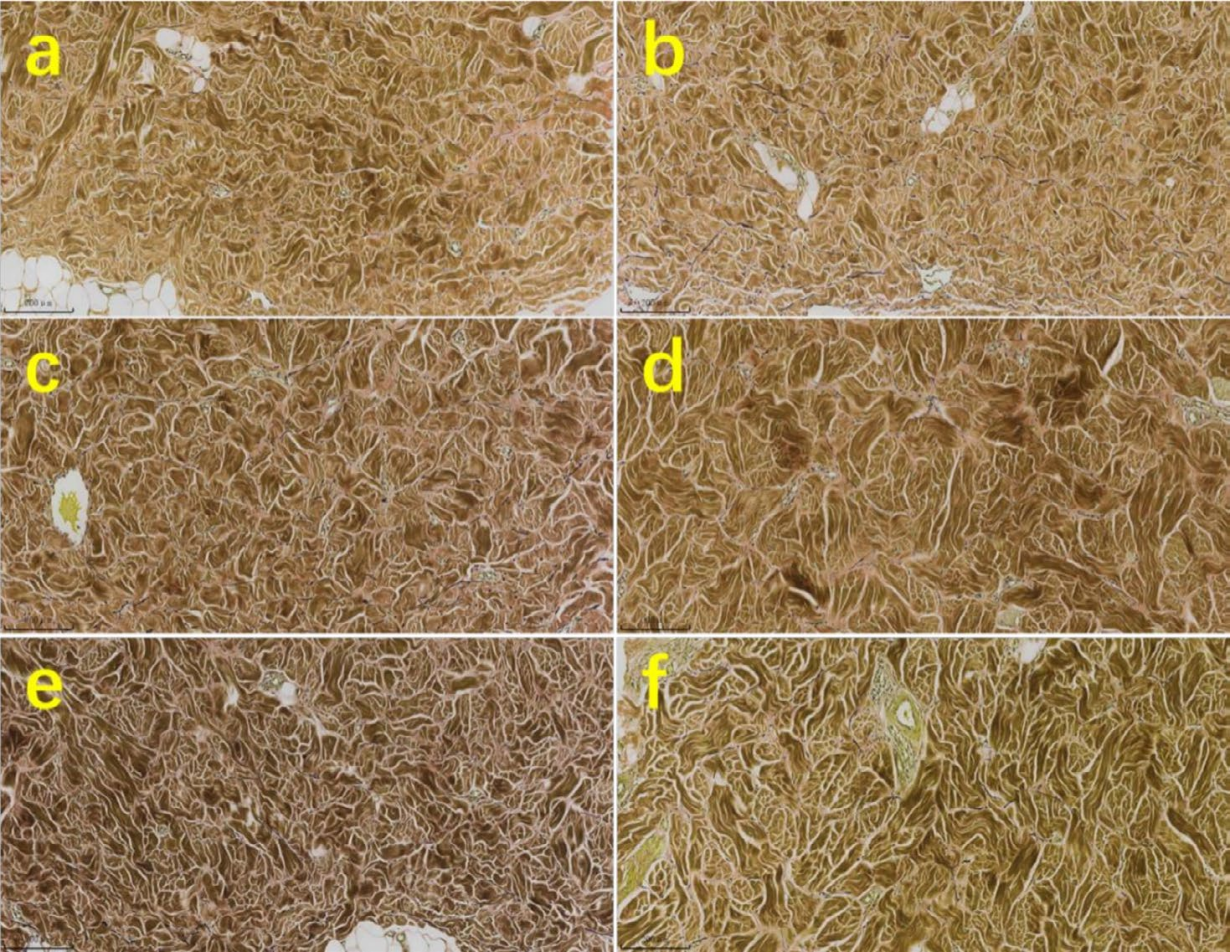
Verhoeff–Van Gieson staining of elastin fibers in the six treatment groups. Histological sections stained with Verhoeff–Van Gieson reveal variations in elastin fiber regeneration across treatment groups: (a) PLLA injection only; (b) MFU treatment only; (c) MFU treatment immediately before PLLA injection; (d) MFU treatment 1 month before PLLA injection; (e) MFU treatment 1 month after PLLA injection; (f) No treatment.

Verhoeff–Van Gieson staining revealed differences in elastin fiber regeneration among the six treatment groups. The untreated group (Figure 5f) exhibited the lowest elastin fiber density, with sparse and irregular fiber distribution. Compared to the untreated group, the PLLA injection‐only group (Figure 5a) demonstrated better elastin fiber regeneration, with increased staining intensity and fiber density. The MFU treatment‐only group (Figure 5b), however, showed marginally less improvement than the PLLA injection‐only group. The three combination treatment groups exhibited superior elastin fiber regeneration compared to the single‐treatment groups. Group c (MFU treatment immediately before PLLA injection) and group d (MFU treatment 1 month before PLLA injection) demonstrated the most prominent improvements, characterized by dense and organized elastin fibers with deep staining (Figure 5c,d). These results indicate that combined PLLA injection with MFU treatments is more effective in promoting elastin fiber regeneration compared to single treatments.

### 3.6 MFU Effects on PLLA Stability and Inflammation

**FIGURE 6:**
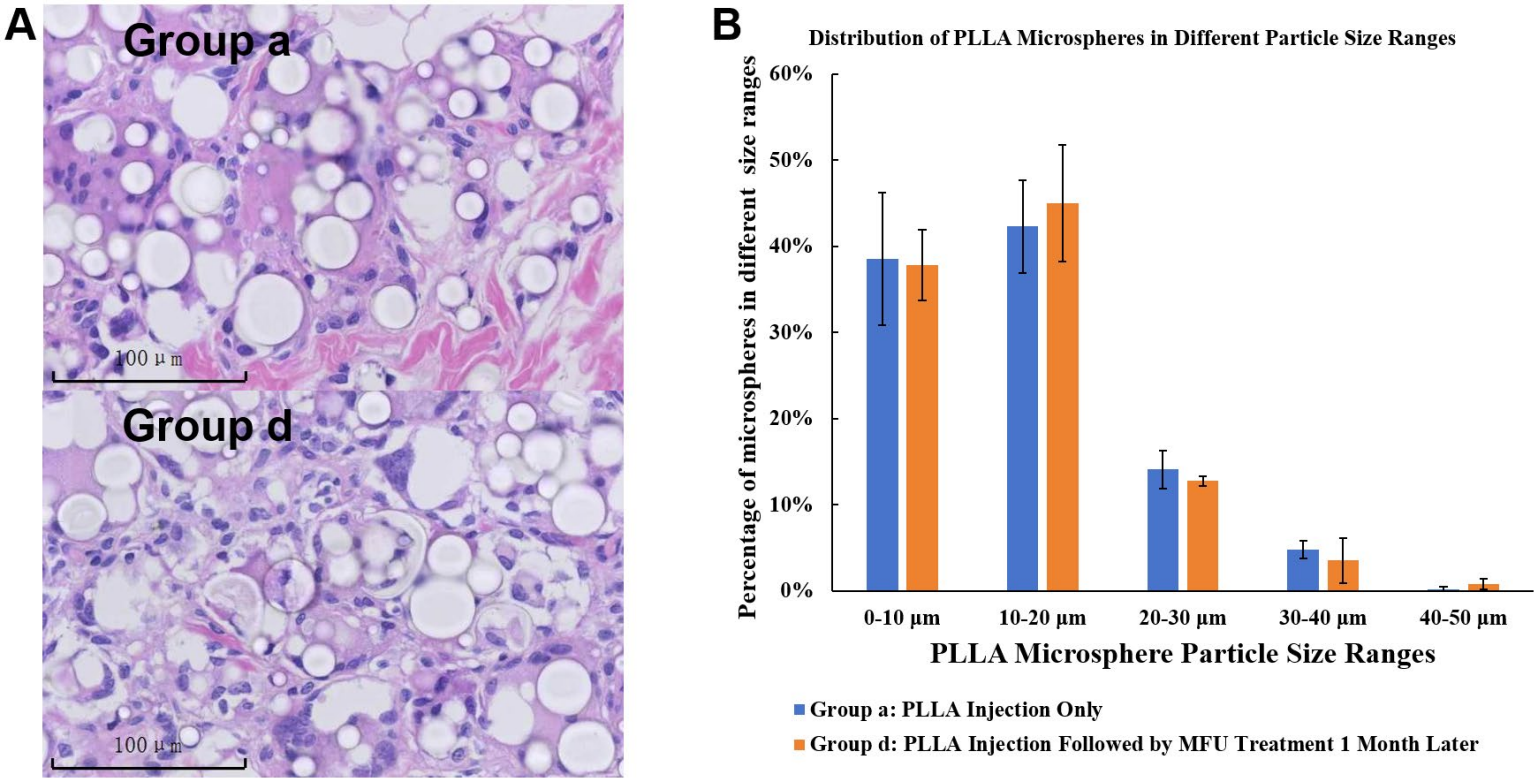
Histological comparison and particle size distribution of PLLA microspheres in Group a (PLLA injection only) and Group d (PLLA injection followed by MFU treatment 1 month later) at 180 days post‐PLLA injection. (A) Representative histological images of Group a (top) and Group d (bottom) stained with hematoxylin and eosin (H&E). The images show PLLA microspheres (white transparent spherical structures) within the tissue at 180 days post‐injection. Scale bar = 100 μm. (B) Distribution of PLLA microspheres in different particle size in Group a (blue bars) and Group d (orange bars). Error bars represent the standard deviation.

HE staining was used to evaluate the stability of PLLA microspheres within the subcutaneous fat layer following treatment. In Group a, representing the PLLA‐only group, the microspheres appear intact, evenly distributed, and surrounded by a mild level of inflammatory cell infiltration, indicative of the biostimulatory response. In Group b, where MFU was administered 1 month after PLLA injection, the microspheres remain structurally stable, with no evidence of accelerated degradation or fragmentation. Additionally, MFU treatment did not exacerbate the inflammatory response induced by PLLA implantation, as the level of inflammatory cell infiltration surrounding the microspheres was comparable to that in the PLLA‐only group (Figure 6A). Furthermore, quantitative analysis shows that the particle size distribution of PLLA microspheres in both groups (Group a and Group d) is similar, with no significant differences observed across the measured size ranges. This indicates that MFU treatment does not alter the microsphere degradation pattern or particle size distribution over the observation period (Figure 6B). These findings demonstrate the compatibility and safety of combining MFU with PLLA for subcutaneous tissue remodeling.

## 4 Discussion

Poly‐l‐lactic acid (PLLA) is a widely utilized medical aesthetic device known for its unique mechanism of action and long‐lasting benefits [18]. Unlike hyaluronic acid (HA), which primarily provides immediate hydration and volumization, PLLA serves as a biostimulator, gradually promoting collagen synthesis in the dermis. This leads to sustained structural improvement and enhanced elasticity over several months [19]. PLLA has been extensively studied and successfully applied in various areas of the body. Research has demonstrated its efficacy in facial rejuvenation, improving skin laxity in the lower face and neck, and addressing volume loss in the midface [20, 21]. Additionally, it has shown promising results in non‐facial applications, such as enhancing the appearance of hands, décolletage, and buttocks [22, 23]. The versatility and biocompatibility of PLLA have made it an integral component of medical aesthetic treatments, catering to a wide range of patient needs and anatomical areas [24].

Energy‐based devices like micro‐focused ultrasound (MFU) complement injectable treatments by inducing thermal effects that stimulate collagen remodeling and elastin production [25]. MFU is particularly effective in promoting type I collagen production and dermal thickening through heat‐induced fibroblast activation in deeper tissue layers, such as the SMAS and reticular dermis [13]. When combined with PLLA, which excels at stimulating type III collagen production in the early phases and sustaining type I collagen deposition over time, the two modalities create a synergistic effect [26]. MFU primes the dermis with thermal coagulation, enhancing PLLA's biostimulatory effects for long‐term remodeling. This layered approach ensures both immediate skin tightening and sustained structural improvements [27].

Our findings in this study demonstrate that integrating PLLA with MFU results in notable advancements in tissue remodeling, surpassing the effects of individual treatments. The sequential application of MFU, especially immediately before PLLA injection, achieved the greatest improvements in dermal architecture, characterized by enhanced collagen and elastin fiber regeneration and well‐structured subcutaneous fat septa. Importantly, PLLA microspheres remained stable following MFU treatment, with no evidence of accelerated degradation or heightened inflammatory response. This confirms the safety and compatibility of combining these two therapeutic modalities.

Although excessive thermal energy was hypothesized to potentially disrupt PLLA microspheres [28], our findings demonstrated no structural instability or accelerated degradation 6 months post‐injection, regardless of MFU application timing. Furthermore, inflammatory cell infiltration levels were comparable between MFU‐treated and PLLA‐only groups, suggesting that the stability of PLLA microspheres under thermal effects is influenced by their micro‐molecular structure and average chain length. These results emphasize the importance of optimizing MFU treatment protocols to balance safety and efficacy.

To ensure preclinical relevance, Bama miniature pigs, known for their dermal similarities to humans in collagen composition, dermal thickness, and adnexal structure, were selected as the animal model [13]. However, we acknowledge that interspecies differences—such as metabolic rate, immune response intensity, and wound‐healing kinetics—may influence the magnitude and duration of tissue remodeling responses. Therefore, the quantitative histological outcomes reported here should be interpreted as mechanistic evidence rather than direct predictors of human clinical efficacy. Nevertheless, the consistent histological patterns observed across treatment groups support the translational potential of this combined approach. Studies involving human subjects will be essential to confirm these findings under clinical conditions and across different skin types and ethnicities, while also elucidating the molecular mechanisms of PLLA and MFU interactions, particularly the role of heat‐induced fibroblast activation.

## 5 Conclusion

This study demonstrates the synergistic benefits of combining PLLA with MFU, with the sequence of MFU immediately prior to PLLA yielding the most significant improvements in dermal thickness, collagen density, and elastin fiber regeneration. Importantly, MFU did not accelerate PLLA microsphere degradation, highlighting its safety and compatibility. These findings provide a foundation for refining clinical protocols, and further research is needed to optimize these combined therapies for broader clinical application.

## Author Contributions

Qiong Wu and Changxin Jin: Conducting experiments, data interpretation, writing – original draft. Shixu Wang: Conducting experiments, data interpretation. Gaoxu Li and Ruixue Li: Conducting animal experiments. Wei Zhan, Yijie Wang, and Bo Zhou: Data collection and analysis. Dan Lin: Supervision, writing – original draft, review and editing. Fulin Chen: Conceptualization, supervision. All authors were involved in the final approval.

## Funding

This work was supported by the Research Funding of Xi'an No. 1 Hospital (The First Affiliated Hospital of Northwest University, Grant 2025001).

## Ethics Statement

This study was approved by the Institutional Animal Care and Use Committee (IACUC) of the College of Life Sciences, Northwest University (Approval No. IACUC‐20241487). All procedures followed ethical guidelines for the care and use of laboratory animals.

## Conflicts of Interest

The authors declare no conflicts of interest.

## Data Availability

The data supporting the findings of this study can be obtained from the corresponding author upon reasonable request.
